# Impact of Electrolyte Temperature on Solution‐Combustion Synthesized NiO/Ni Nanoparticles as Oxygen Evolution Reaction Electrocatalyst

**DOI:** 10.1002/open.202500137

**Published:** 2025-11-02

**Authors:** Easwari Padma Kumari, Anand Kumar, Faris Tarlochan, Mohammed J. Al‐Marri

**Affiliations:** ^1^ Department of Mechanical and Industrial Engineering College of Engineering Qatar University P O Box 2713 Doha Qatar; ^2^ Department of Chemical Engineering College of Engineering Qatar University P O Box 2713 Doha Qatar; ^3^ Gas Processing Center College of Engineering Qatar University P O Box 2713 Doha Qatar

**Keywords:** electrothermal catalysis, elevated temperature water splitting, nickel oxide, oxygen evolution reactions, solution combustion synthesis

## Abstract

Oxygen evolution reaction (OER) is an important half‐cell reaction in water electrolysis; however, its sluggish kinetics and high overpotential limits efficiency, require highly active and stable catalysts. This study explores the effect of electrolyte temperature on the OER activity of NiO/Ni catalyst synthesized via solution combustion synthesis. Results reveal a remarkable reduction in overpotential from 550 to 356 mV, along with a significant increase in current density, demonstrating the impact of electrolyte temperature on OER kinetics. The Tafel slope decrease progressively, reaching 75.8 mV dec^−1^ at 30 °C, indicating improved reaction kinetics and charge transfer efficiency. Additionally, the increase in double‐layer capacitance (*C*_dl_) with temperature confirms greater exposure of electrochemical surface area, providing more active sites for the reaction. Stability tests over 1000 CV cycles confirmed excellent durability, making NiO/Ni a highly efficient catalyst for alkaline OER. These findings highlight the electrolyte temperature optimization as an effective approach to improving catalytic performance of NiO/Ni to act as a promising material for cost effective sustainable energy applications.

## Graphical abstract

Electrolyte temperature significantly affects electrocatalyst performance in water splitting reactions. Catalysts synthesized by solution combustion synthesis were evaluated for oxygen evolution activity at different temperatures. Elevated temperatures reduced overpotential and increased current density, reflecting enhanced reaction kinetics and efficiency. Since overpotential indicates energy loss and sluggish reaction steps, its reduction demonstrates improved catalytic activity. Thus, the electrolyte thermal conditions critically affects water splitting.

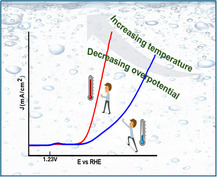

## 1 Introduction

In the global struggle for economic growth, environmental protection is a priority; fossil fuels serve as a symbol of human innovation and a reminder of the challenges ahead in building a sustainable future. The combustion of these substances is believed to directly disturb climate change and contribute to global warming.^[^ ^1^ ^]^ The extraction and processing of fossil fuels often results in adverse impacts on ecosystems, water sources, and local communities, manifesting in habitat destruction, pollution, and health risks.^[^ ^2^ ^,^ ^3^ ^]^ As finite resources, fossil fuel resources face depletion, posing challenges to long‐term energy security and economic stability. At present, hydrogen, recognized as a clean and versatile energy carrier with the potential to play a central role in future sustainable energy systems. It can be generated through a variety of production routes, including conventional fossil‐based methods such as steam methane reforming, coal gasification, and partial oxidation of hydrocarbons, as well as emerging processes like urea electrolysis and biomass gasification.^[^ ^4^, ^5^, ^6^, ^7^, ^8^ ^–^ ^9^ ^]^ Electrochemical methods reduce the dependance on nonrenewable energy sources and provide efficient energy conversion, enabling clean energy production. These technologies provide an easier path toward a more sustainable approach and assure a better energy future for generations to come. Using an electrochemical process, fuel cells convert fuel energy into electrical energy, representing an innovative technology with minimal environmental impact.^[^ ^10^ ^,^ ^11^ ^]^ Water electrolysis is currently the primary method for producing massive quantities of hydrogen, but this method faces significant challenge, in terms of cost and energy consumption.^[^ ^12^ ^,^ ^13^ ^]^ The energy required for electrolysis, which is related to cell voltage, presents a major barrier to its efficiency and expenses. The cell reactions that produce hydrogen and oxygen during water splitting are known as the hydrogen evolution reaction (HER) and the oxygen evolution reaction (OER). These reactions are redox reactions with specific thermodynamic potentials. A potential of 1.23 V is required for water splitting, known as the theoretical voltage of water electrolysis. The voltage is associated with the energy required for HER and OER at their respective electrodes.^[^ ^14^ ^,^ ^15^ ^]^ Protons (H^+^) are reduced in HER to create hydrogen gas (H_2_), which has a theoretical voltage of about 0 V, under standard conditions and OER produce oxygen gas (O_2_), involves the oxidation of water molecules has a theoretical voltage of ≈1.23 V under the same conditions.^[^ ^14^ ^,^ ^15^ ^]^ Therefore, to generate hydrogen efficiently, the applied cell voltage must exceed the thermodynamic threshold of 1.23 V to overcome kinetic barriers and system losses. Overvoltage is needed to compensate for the losses caused by kinetic limitations and the ohmic resistance of the electrolyte.^[^ ^16^ ^]^ This ensures that sufficient energy is provided to drive both reactions in the desired direction, leading to the decomposition of water into respective gases. Electrocatalysts that promote electrochemical processes can increase the efficiency of water electrolysis. Thus, by reducing the energy loss caused by slow kinetics, these catalysts contribute to more effective methods for converting and storing energy. To improve the overall functionality and energy efficiency of electrolysis technology, efficient OER is crucial. The development of advanced catalysts for OER is critical because they can significantly lower energy barriers, improve reaction kinetics, and reduce operational costs. Effective OER catalysts not only enhance the economic feasibility of hydrogen generation but also affect the scalability and integration of renewable energy sources. OER is a sluggish reaction involving a multistep four‐electron transfer process. The adsorbate evolution mechanism (AEM) describes the OER pathway in which hydroxyl ions (OH^−^) from the alkaline electrolyte are sequentially oxidized on the catalyst surface to form adsorbed intermediates, leading to the evolution of molecular oxygen and water. Here, water molecules or hydroxyl ions interact with transition metal active sites, M(*). A hydroxyl ion from the electrolyte is adsorbed onto the metal site, forming an M–*OH intermediate through a one‐electron oxidation step (surface adsorption). This intermediate undergoes coupled proton and electron removal to yield the M–*O species. This step is often associated with significant energy barriers due to strong *O binding. This then reacts with another OH^−^ or H_2_O molecule to form M–*OOH via O—O bond formation. This is typically the rate‐determining step (RDS) due to high Δ*G* value. In the final step*,* M–*OOH undergoes one‐electron oxidation to release molecular oxygen (O_2_), regenerating the original metal active site. Each of these steps involves electron and proton transfer events, and the efficiency of the overall process is strongly influenced by the ability of the catalyst to stabilize these intermediates and facilitate charge transfer.
(1)

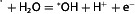



(2)

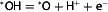



(3)

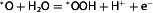



(4)

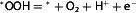




AEM involves OH_ads_, O_ads_, OOH_ads_, and O_2ads_. The adsorption energies of reaction intermediates serve as an important indicator of electrocatalyst activity. In the four‐step OER pathway (Equation (1)−(4)), each intermediate is associated with a Gibbs free energy change (Δ*G*), and the step with the largest Δ*G* is identified as the RDS, determines the overall reaction kinetics.^[^ ^17^ ^,^ ^18^ ^]^ According to the Sabatier principle, an efficient catalyst achieves a balance in binding strength—if the adsorption is too strong, intermediates accumulate and hinder subsequent steps, whereas too weak adsorption prevents their stabilization. Thus, the optimal OER catalyst minimizes the free‐energy barrier of the RDS by binding intermediates with moderate strength, leading to lower overpotential and enhanced catalytic activity. For the OER to take place, the applied potential must be greater than the equilibrium half‐cell potential so that the products are thermodynamically more stable than the reactants. The additional driving force beyond the equilibrium potential is defined as the overpotential (*η*). The binding energy of intermediates influences this process. If an intermediate adsorbs too weakly (Δ*G* > 0), the initial adsorption step becomes energetically uphill, strong binding (Δ*G* < 0) makes the following step energetically unfavorable. Thus, the optimal catalyst must provide an intermediate binding strength, ensuring that no single step dominates the reaction pathway and thereby minimizing the overall overpotential. The electronic structure of Ni/NiO composites enhances the electrical conductivity and fine‐tunes the adsorption strength of oxygen evolution intermediates. This effect reduces the energetic requirements for the reaction and speeds up the overall kinetics. Together these two phases provide a favorable pathway for rapid charge transport and intermediate stabilization, resulting in superior OER activity. Comparable behaviors have been reported in previous computational investigations on related Ni‐based heterostructures, where interfacial engineering was found to promote electron mobility, balance adsorption energies of key intermediates in OER activity.^[^ ^19^, ^20^ ^–^ ^21^ ^]^ Oxides of iridium, ruthenium (Ir, Ru) are known for its high effectiveness in catalytic activity toward OER.^[^ ^22^, ^23^, ^24^, ^25^, ^26^, ^27^ ^–^ ^28^ ^]^ But the excessive cost of noble metals raises expense, hindering large‐scale use, particularly in cost‐sensitive applications. The expensive noble metal catalysts can be replaced by catalysts that use transition metal oxides and perovskites containing iron (Fe), nickel (Ni), cobalt (Co), manganese (Mn), and molybdenum (Mo).^[^ ^29^, ^30^, ^31^ ^–^ ^32^ ^]^ Nickel is an important metal that exhibits high stability in alkaline environments, depends on M—H bond strength.^[^ ^33^ ^]^ They are the best choice for low‐cost electrode materials due to their earth abundance and capability to operate efficiently at high current density. The favorable electronic properties contribute to its catalytic performance by facilitating interactions with reactants and intermediates. Ni has been shown to benefit from the synergistic effects when combined with other metals by stabilizing Ni^2+^/Ni^3+^ redox couples, improving their overall performance and stability for large‐scale applications.^[^ ^34^ ^,^ ^35^ ^]^ Nickel oxides can undergo potential‐dependent phase transitions, where the surface is reversibly converted into an OER‐active oxyhydroxide layer under oxidative conditions, which results in their high catalytic performance.^[^ ^36^ ^]^ The electrochemical reaction of the catalysts was improved by different NiO morphologies studied by different synthesis method.^[^ ^37^, ^38^ ^–^ ^39^ ^]^ Numerous studies have reported the temperature impact on the catalytic action of oxygen evolution reaction. Temperature‐dependent OER studies can provide details on the kinetics of the reaction and the electron transfer processes. Alkaline water electrolysis (AWE) has been applied industrially to produce hydrogen as a good carbon neutrality method. To make AWE more cost‐effective, extensive research has focused on developing suitable electrocatalysts for the HER and OER. The electrolyte in large‐scale AWE systems is maintained within a temperature window (50–80 °C), meaning that room‐temperature performance data provide limited guidance for catalyst selection in real applications. So it is very important to evaluate how electrolyte temperature affects electrocatalyst activity to design catalyst materials for practical use. Both Eyring equation, $k=\frac{K_{B}T}{h}\exp\;\left(-\frac{\text{Δ}G^{*}}{RT}\right)$ and Arrhenius equation $k=\text{A}\text{exp}\left(-\frac{\text{E}a}{RT}\right)$ explains the thermodynamically and kinetically temperature dependency on the rate of the half‐cell reactions in water electrolysis,^[^ ^40^ ^,^ ^41^ ^]^ Also, temperature has influence on the key parameters on the electrochemical water splitting reactions including mass transport and adsorption of species in the OER process. Thus, there is a need to treat electrolyte temperature not as a background variable, but as a design parameter for tuning electrocatalyst behavior in practical AWE. The OER activity of Ni—Co‐based catalysts in alkaline conditions across different temperature ranges showed an increasing trend in current density.^[^ ^42^ ^]^ Corina et al. reported the importance of catalyst stability study under realistic conditions for industrial applications by evaluating the NiFe layered double hydroxide (LDH) as OER active catalyst.^[^ ^43^ ^]^ The increased exchange current density value of the nickel oxide nanostructures (nanoNiOx/GC) synthesized at a higher temperature than the room temperature was found to decrease the particle size.^[^ ^44^ ^]^ The reduction in particle size and the concentration of electroactive surface species resulted in enhanced OER kinetics due to an increase in the preparation temperature in 0.5 M KOH.^[^ ^44^ ^]^ The morphology of the catalyst also facilitates the increased gas evolution reaction. The electrochemical activation energy (*E*_A_) for the OER varies with temperature, with values of 31.6 kJ mol^−1^ for unmodified nickel foam and 54.5 kJ mol^−1^ for platinum‐activated nickel foam, indicating that temperature affects the energy barrier for the reaction.^[^ ^45^ ^]^ This study examines how the electrolyte temperature affects the solution combustion synthesis (SCS)‐derived NiO/Ni catalyst's electrocatalytic OER performance. The prepared catalyst was evaluated electrochemically on a glassy carbon electrode in 1 M KOH. Characterization techniques like X‐ray diffraction (XRD), scanning electron microscopy (SEM), and transmission electron microscopy (TEM) was used to understand about the material's structure, morphology, and composition which affects the catalyst's activity. By measuring the binding energies of core‐level electrons, X‐ray photoelectron spectroscopy (XPS) helps in understanding the oxidation states and nature of chemical bonds present on the surface, which are crucial for optimizing the catalyst's performance in OER applications. A linear sweep voltammetry (LSV) studies electrochemical properties, while cyclic voltammetry (CV) sweeps the potential between two values to observe redox processes. The electrochemical surface area (ECSA) represents the active surface area available for reactions, which is crucial for catalytic activity. Double‐Layer Capacitance (*C*_dl_) measures the capacitance of the electrical double layer at the electrode–electrolyte interface and is often used to estimate the ECSA. Together, these techniques provide details into the electrochemical behavior and surface properties of catalysts. The stability of a catalyst determines how well it maintains activity after extensive use, which is crucial for practical applications.

## 2 Experimental Section

### 2.1 Materials Required

All materials used in the experiments are of analytical grade. Nickel nitrate hexahydrate [Ni(NO_3_)_2_ · 6H_2_O, crystallized, ≥97%], the metal nitrate, and glycine [C_2_H_5_NO_2_], which was used as the fuel of the combustion process, isopropyl alcohol (C_3_H_8_O), Nafion solution (5 wt%) all were from Sigma–Aldrich. Carbon black was purchased from Cabot corporation and potassium hydroxide (KOH) was obtained from Qualikems. Deionized (DI) water was used to prepare the solution.

### 2.2 Catalytic Synthesis and Ink Preparation

**Figure 1:**
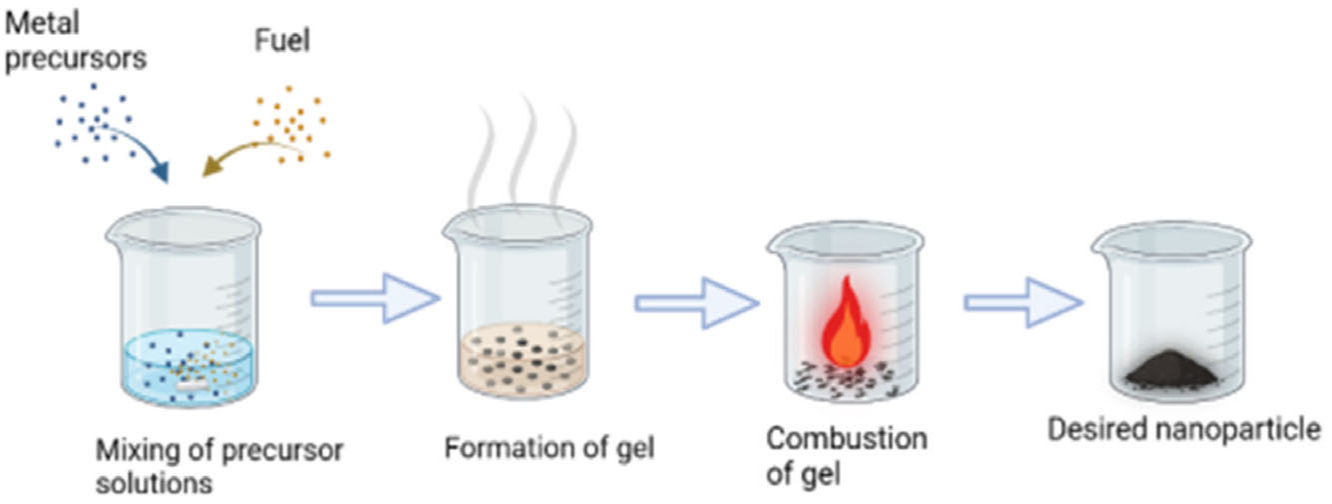
Schematic showing the catalyst synthesis method.

NiO/Ni was synthesized using SCS method. This cost‐effective SCS can be used to synthesize transition metal catalysts with high surface area and porous structures with high catalytic activity as well as their oxide forms.^[^ ^46^, ^47^ ^–^ ^48^ ^]^ The combustion reaction of glycine as fuel with Ni‐based materials are caused by the gas phase exothermic reaction between HNO_3_ and NH_3_, the decomposition products of the precursors itself.^[^ ^49^ ^]^ A diagram of the synthesis method is shown in **Figure** **1**, created with BioRender.com accessed on August 19, 2024 https://www.biorender.com. The, Ni(NO_3_)_2_ · 6H_2_O and glycine (C_2_H_5_NO_2_) were measured based on the stoichiometric equation with fuel ratio, *φ *= 1. The precursors were dissolved in DI water and stirred for 15 min to achieve a visually uniform solution. This solution was then heated using a hot plate with stirring to get a viscous gel. With continued heating above 200 °C the gel expanded, and auto ignited, when reached the ignition temperature, it generated a black powder. The powder was crushed using a hand mortar.

The synthesized catalyst of 40 mg was added with 60 mg of carbon black, to make it conductive during electrochemical reactions. This was distributed in 10 mL of deionized water and well sonicated for about 1 h. The prepared catalyst‐carbon solution was then heated to remove the water content in it. The sample obtained was grinded to get uniform particles.

### 2.3 Characterization Techniques

Synthesized catalyst's phase(s) was identified using a PANalytical EMPYREAN X‐ray diffractometer (XRD) at a wavelength of 0.154056 nm of Cu‐K*α* radiation scanned at 10°–90° (2*⊖* range). SEM analysis was conducted to understand sample morphology using a Nova Nano SEM 450. TEM (Tecnai G2 TF20 model of FEI machine) with a 200‐kV acceleration voltage was used to analyze the nanoscale structure of the synthesized catalyst.

### 2.4 Electrochemical Experiment

A fixed amount of the catalyst, 5 mg was mixed with 300 μl of isopropanol and 30 μl of Nafion solution, serves as a binder to enhance film adhesion and ionic conductivity. The mixture was ultrasonicated for 30 min in a temperature‐controlled bath to ensure homogeneous dispersion of the catalyst particles. Maintaining a lower temperature during sonication helps prevent solvent evaporation and thermal degradation of the ink components, thereby preserving ink stability. Ultrasonication is a widely adopted method to break up agglomerates and achieve homogeneity, with durations typically ranging from 15 to 60 min.^[^ ^50^ ^]^ Predispersing the catalyst in the solvent before adding the binder helps to minimize aggregation and improve ink stability. Visual inspection of the ink, along with its spreading behavior during drop‐casting, can offer practical confirmation of uniform dispersion. The ink was used immediately after dispersion to avoid sedimentation. From the obtained ink, 3 μl ink was dropped to a 5 mm glassy carbon disc, which was mounted on the tip of an electrode rotator (Wave Vortex 10 Electrode Rotator, PINE instruments) and dried at room temperature with a rotation of 250 rpm for 1 h 30 min. A solution of 1 M KOH was used for conducting electrocatalytic experiments, which was fully purged prior to the experiments with nitrogen gas for 30 min to remove any gaseous impurities in the electrolyte. Electrochemical temperature‐dependent measurements were conducted using a rotating disk electrode (RDE) setup integrated with a double‐jacketed electrochemical cell. The outer jacket was connected to a circulating water bath thermostat, which maintained the desired electrolyte temperature by continuously circulating water around the cell chamber. The temperature of the electrolyte inside the working compartment was monitored and controlled using a thermostat probe, ensuring stable and uniform thermal conditions throughout the experiment. Prior to each measurement, the system was allowed to stabilize for 10–15 min to ensure that the electrolyte and electrode reached the target temperature. All measurements were performed under isothermal conditions, and the temperature was periodically verified to avoid errors. This study was conducted at four different temperatures from 10 °C, 20 °C, 25 °C to 30 °C. The tip of the RDE with a glassy carbon electrode attached was immersed in the electrolyte. All potentials were measured using standard Ag/AgCl (4 M saturated KCl) reference electrode and Pt coil as the counter electrode. This N_2_ saturated electrolyte was used to pretreat the working electrode for 100 reduction/oxidation cycles at a scan rate of 500 mV s^−1^ at 1600 rpm to activate and clean the electrode surface. This precycling ensures that the system reaches a stable state. The LSV polarization curve was scanned at a scan rate of 10 mV s^−1^, repeated 3 times from 0 to 1.0 V (vs. Ag/AgCl). This electrode was then analyzed with the CV under different scan rates (20, 40, 60, 80, 100 mV s^−1^) to study the redox property of the catalyst in the same potential window under temperature. The current values were converted into current density by dividing with an area of 0.196 cm^2^ of working electrode. Voltages in Ag/AgCl, then converted to reversible hydrogen electrode (RHE) values using Equation (5) and used for plotting the data.
(5)

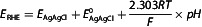

where, E(Ag/AgCl)—measured potential; 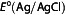
—the standard potential of the Ag/AgCl electrode at the specific temperature; *R*—gas constant; *T*—temperature in Kelvin; and *F*—Faraday constant.

By analyzing the current response in the nonFaradic zone, double‐layer capacitance (*C*_dl_) can be estimated. The *C*_dl_ of electrodes was estimated at varied scan rates (20, 40, 60, 80, 100, 150, and 200 mV s^−1^). The double‐layer charging current was graphed against the CV scan rate, and the slope of linear regression was used to determine the slope value. All the above‐mentioned procedures were performed at all temperatures under study which was controlled by a circulating bath purchased from PolyScience.

## 3 Results and Discussion

### 3.1 X‐ray diffraction (XRD)

**Figure 2:**
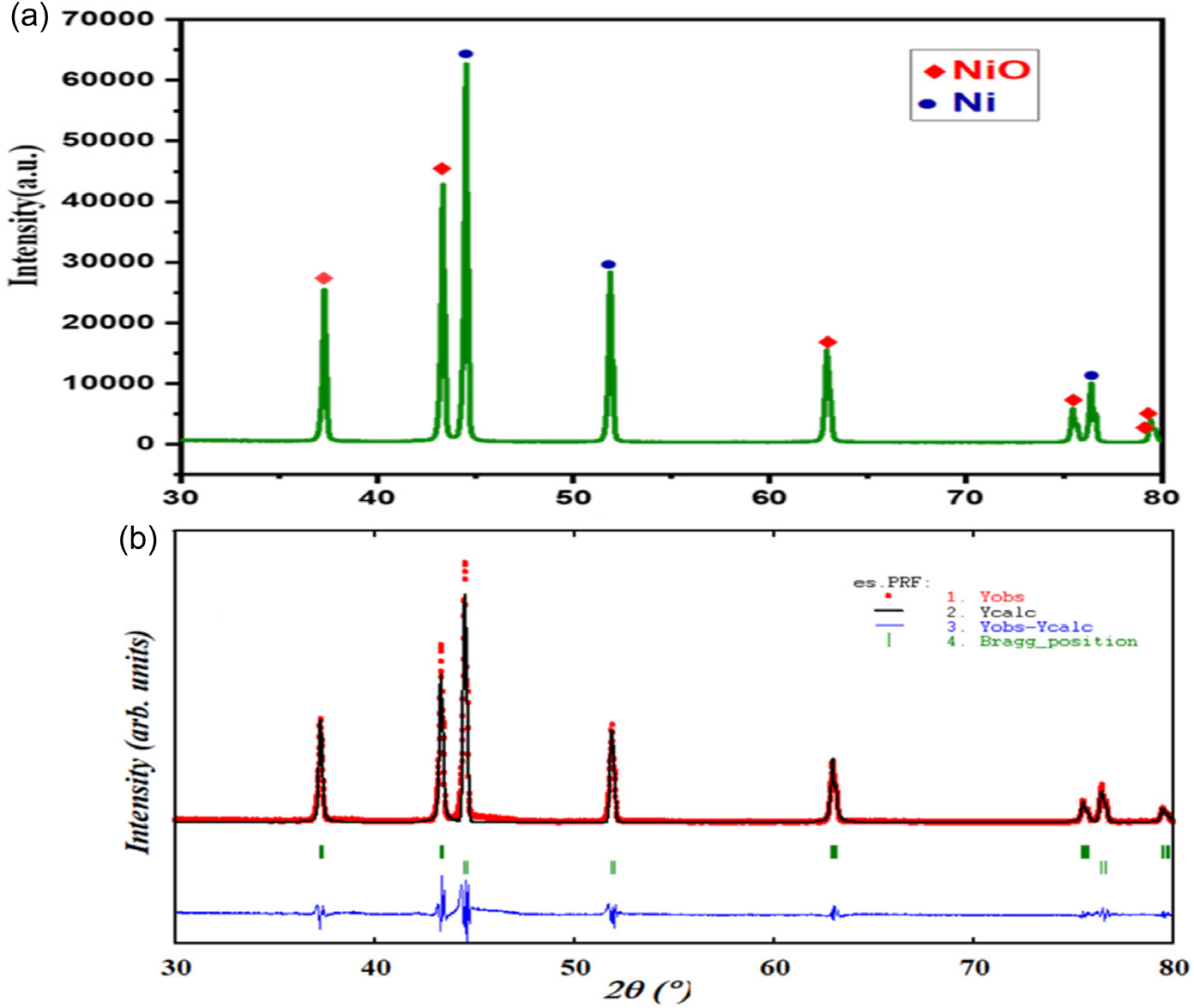
a) XRD characterization peaks of synthesized catalyst and b) Rietveld refinement plot of the prepared sample showing the experimental, calculated, and difference pattern.

To characterize the crystalline phases and purity of the catalyst synthesized by solution combustion, XRD examination was performed. The XRD pattern shown in **Figure** **2**a revealed distinct peaks corresponding to both cubic NiO and Ni phases. The pattern displayed at 2*θ* angles of 37.29°, 43.32°, 62.87°, 75.42°, and 79.41° corresponded to the (111), (200), (220), (311), and (222) and at 44.51°, 51.9°, and 76.39° corresponded to (111), (200), and (220) for NiO and Ni, respectively, in agreement with standard data JCPDS no. 04–0835 and JCPDS no. 065–0380.^[^ ^51^ ^,^ ^52^ ^]^ The excessive pressure generated results in insufficient oxygen, favoring the formation

of metallic Ni.^[^ ^52^ ^]^ The (111) peak of Ni exhibited significantly higher intensity compared to the corresponding peaks of NiO, indicating a concentration of cubic Ni phase formation in the synthesized sample. Incorporation of metallic Ni atoms decreases the bandgap of NiO, which facilitates faster charge transfer at the electrode–electrolyte interface.^[^ ^53^ ^]^ The catalyst's crystallite size (*D*) was calculated using Scherrer's formula based on the full width at half maximum (FWHM) of the diffraction peak (*β*),

(6) D=kλ/βcosθ

where ”*λ*“ is the wavelength of the X‐ray (1.541 Ao), ”*θ*“ is the Bragg's angle, and "*k"* is a constant depending on the shape of the grain (0.94). The Scherrer calculation shows an average crystallite size of 29 nm for the synthesized catalyst. The fraction of the observed phases as quantified by Rietveld refinement was found to be 52.97% of NiO and 47.03% of Ni in the sample (Figure 2b).

### 3.2 Scanning electron microscopy (SEM)

**Figure 3:**
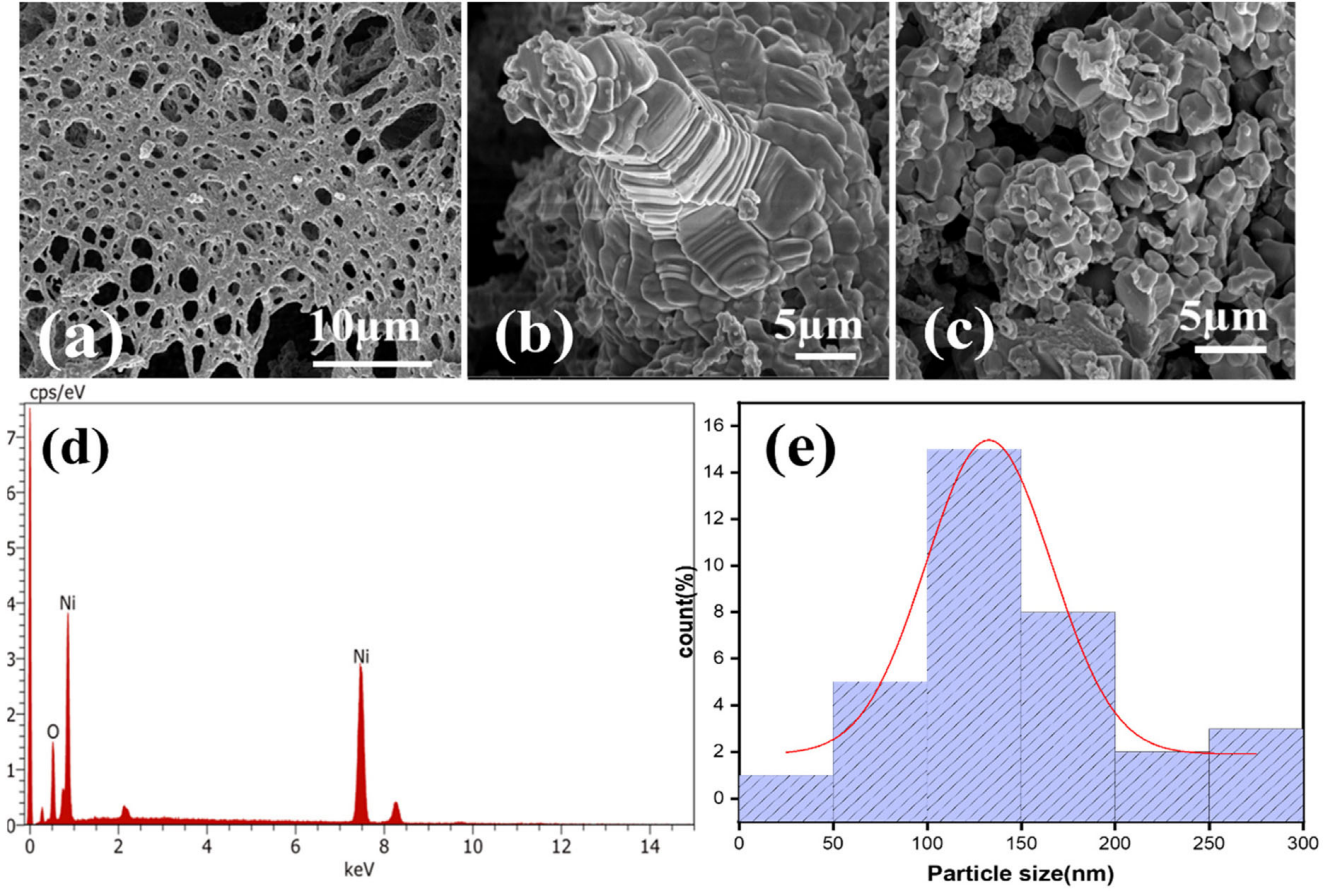
a) SEM image at a lower magnification, b,c) SEM image at a higher magnification, d) EDS spectrum showing elements present in the catalyst Ni/NiO, and e) histogram showing the particle size distribution.

**Figure** **3**a displays a porous foam‐like structure from the synthesized catalyst's SEM micrographs at low magnification. Combusted materials have porous structures as an outcome of gas production during the combustion process.^[^ ^54^ ^]^ This porous and connected morphology facilitates the diffusion of reactants and products throughout the material, improving mass transport and maintaining high reaction rates during OER. But at a higher magnification images Figure 3b,c, show that the foam walls are composed of agglomerated particles, suggests the porous walls are constructed from densely packed nanoscale domains*.* The (111) diffraction peak of Ni in the XRD pattern, characteristic of its face‐centered cubic (FCC) phase, indicates a high abundance of metallic Ni in the bulk, likely comprising Ni cores with an oxidized NiO surface layer. The aggregation of Ni‐rich domains contributes to the increased intensity of this peak.The energy dispersive X‐ray spectrum (EDS) in Figure 3d verifies the elemental composition of the prepared NiO/Ni catalyst, supporting the coexistence of Ni and oxygen in the structure. Figure 3e presents the particle size distribution of the Ni/NiO catalyst obtained from SEM image analysis. The histogram shows that most particles fall within the 100–150 nm range, with an average size of ≈132 nm. The red fitted curve indicates a near‐Gaussian distribution, suggesting relatively uniform particle growth with some degree of aggregation. The presence of larger particles (>200 nm) can be attributed to agglomeration during high‐temperature calcination. This distribution further supports the SEM observations of a porous, foam‐like morphology composed of polycrystalline aggregates.^[^ ^55^ ^]^ Aggregation increases particle size, reduces surface area by hiding the active area, reduces activity.^[^ ^56^ ^]^ But formation of abundant grain boundaries, Ni/NiO heterointerfaces, and defect‐rich domains that act as additional active sites also generates. These structural features, combined with the porous framework, are expected to enhance charge transfer, facilitate OH^−^ adsorption, and improve overall OER activity. The coexistence of porous structure and nanoscale agglomeration can synergistically enhance electrochemical performance by balancing accessibility, conductivity, and active site density. The interaction between OH^−^ ions and the electrode is a fundamental part of the reaction, influencing its reaction kinetics and efficiency. The porous morphology of nanoparticles enhances this interaction and increases the surface area, which in turn provides more active sites for catalytic reactions, which is crucial for OER, where adsorption and desorption of products takes place.^[^ ^52^ ^,^ ^57^ ^]^ Porous structure improves gas evolution during OER by lowering the charge and mass transfer resistance and reduces the energy barrier required for O_2_ molecule desorption.^[^ ^58^ ^]^ The combination of the metallic Ni and NiO phases create synergistic effect that improves the overall catalytic performance.^[^ ^21^ ^]^ The metallic Ni phase plays a crucial role in improving the material's conductivity by forming a conductive network that facilitates efficient charge transfer, while NiO provides numerous active sites essential for catalyzing the OER, reducing the energy barrier required for oxygen evolution. The presence of an agglomerated phase supports this synergistic effect by enhancing conductivity and creating an optimal environment for NiO to function effectively as a catalyst. This combined action and morphology as suggested by SEM analysis results in improved charge transfer and thus increased OER activity, which is further proved by the study of effect by electrolyte temperature in this work.

### 3.3 Transmission electron microscopy (TEM)

**Figure 4:**
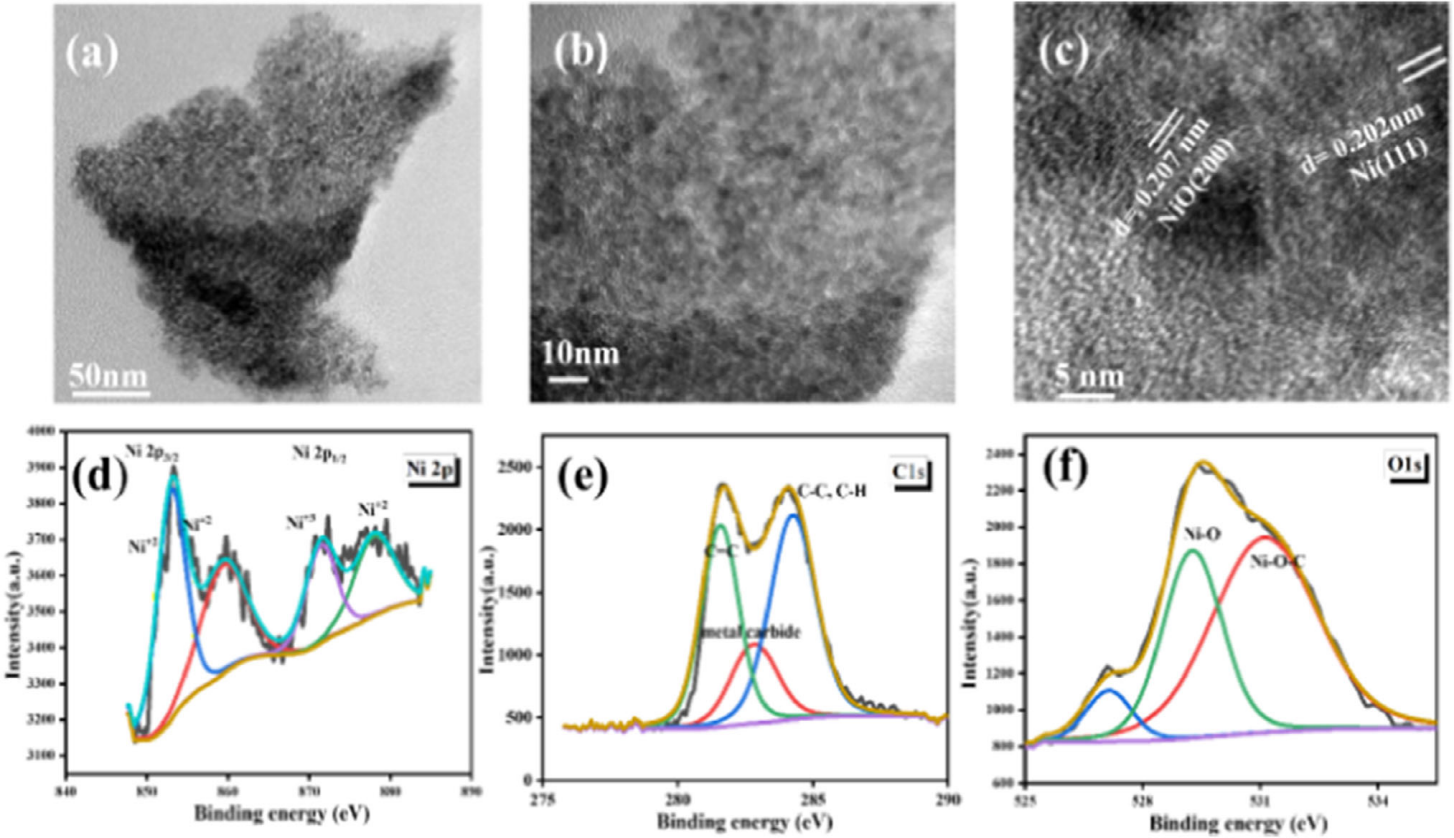
TEM images of the NiO/Ni catalyst showing, a) the agglomeration in the catalyst, b) irregular and fuzed particles, c) HRTEM from a particular area of the catalyst showing interplanar spacing of phases present, and d–f) XPS spectra of NiO/Ni catalyst.

The morphology of NiO/Ni catalyst was further examined using TEM. As shown in **Figure** **4**a, the TEM imaging revealed significant particle agglomeration, making it difficult to distinguish individual particles. Figure 4b displays irregular, fuzed particle shapes, indicating that the particles not only clustered but also merged to form larger, nonuniform domains. This agglomeration behavior is consistent with observations from SEM, reinforcing the material's tendency to form dense clusters. To gain deeper insights into the material's structure, high‐resolution transmission electron microscopy (HRTEM) was performed on selected regions of the sample, as shown in Figure 4c. The HRTEM images revealed lattice fringes with d‐spacing values of 0.202 and 0.207 nm, corresponding to the Ni(111) and NiO(200) crystallographic planes, respectively, confirm the coexistence of both metallic Ni and NiO phases within the catalyst. The presence of mixed phases suggests a complex interplay between Ni and NiO, which may contribute to the material's properties and application toward OER.

### 3.4 X‐ray photoelectron microscopy (XPS)

To determine the oxidation state and elemental composition, high‐resolution XPS of the catalyst material was done. All recorded XPS spectra were analyzed by using casa XPS software. The elements Ni, C, and O were clearly detected by XPS, as shown in Figure 4d–f. Peaks centered at 859, 871.1, and 877 eV were observed, which were separately matched to the binding energy of Ni 2p(3/2) and Ni 2p(1/2).^[^ ^59^ ^]^ The Ni 2p spectra reveals the presence of nickel in the oxide form, characterized by peaks corresponding to Ni 2p_3/2_ and Ni 2p_1/2_ with Ni^+2^ and Ni^+3^ oxidation states, suggest surface oxidation. The Ni(0) peak is not clearly observed in the XPS spectra, although it is detected in the XRD patterns. The possibility of Ni^0^ being embedded within or dispersed throughout the NiO matrix limits its XPS detectability.^[^ ^53^ ^]^ Due to the surface‐sensitive nature of XPS, the signal detected is dominated by NiO, effectively hiding the underlying metallic Ni. This is consistent with SEM observations, where agglomerated domains contain metallic Ni. The presence of surface NiO undergo reversible redox transitions to Ni(OH)_2_ and NiOOH under alkaline conditions. These surface phases modulate catalytic activity, in OER, by facilitating electron transfer and enhancing active site accessibility. The Ni^0^ core may serve as a conductive backbone, promoting charge transport. Thus, the coexistence of bulk Ni^0^ and surface Ni^2+^/Ni^3+^ species provides a synergistic framework for catalytic performance, balancing electronic conductivity with active surface chemistry. The XPS spectrum of C1s shows three carbon peaks at 284 eV corresponding to C=C/C—H. The binding energy ranges from 281.3 eV (C=C) and 285 eV shows the presence of metal carbides in the sample.^[^ ^60^ ^,^ ^61^ ^]^ The XPS analysis of the O 1*s* spectrum of the NiO sample revealed two prominent peaks. These peaks correspond to Ni—O (lattice oxygen) and Ni—O—C bonding, indicating the presence of both types of bonds in the sample.^[^ ^52^ ^,^ ^62^ ^]^ Additionally, an unusual binding energy of 525.8 eV was observed for the O 1*s* spectrum. This value is lower than the typical binding energy of 528 eV for O 1*s* in metal oxides. The observed low binding energy is referred to as the "double charge" effect, which can occur under certain conditions during the XPS measurement.^[^ ^63^ ^]^ This effect resulted in an apparent shift to lower binding energies, providing an explanation for the unusual value observed in the spectrum. These findings highlight the complex chemical environment of the synthesized NiO/Ni sample, with contributions from both metal–oxygen and metal–carbon bonding.

The enhanced OER performance of mixed‐phase Ni/NiO catalysts arises from a synergistic interplay between morphological and electronic features.^[^ ^64^ ^]^ The metallic Ni^0^ core serves as a highly conductive backbone, facilitating rapid electron transport and reducing charge–transfer resistance, which in turn supports efficient redox cycling of surface species. Surrounding this core, the NiO and its derivatives (such as Ni(OH)_2_ and NiOOH) undergo reversible redox transitions under alkaline conditions, providing dynamic and redox‐active sites directly involved in the OER mechanism, particularly in the formation and release of oxygen intermediates. At the NiO/Ni interface, electron‐rich zones and interfacial strain fields emerge, modulating the adsorption energies of key intermediates like OH^−^ and OOH^−^, thereby lowering the energy barrier for water oxidation and enhancing reaction kinetics. Additionally, the mixed‐phase architecture introduces defects, grain boundaries, and heterojunctions, which contribute to a higher density of catalytic sites and increased electrochemically active surface area (ECSA). The presence of metallic Ni within the NiO matrix appears to enhance the catalytic performance.^[^ ^65^ ^]^ The OER activity of Ni‐based catalysts can be explained by the formation of high‐valence Ni species, which serve as the actual active sites for oxygen evolution. In Ni/NiO composites, the metallic Ni phase facilitates electron transport, while the NiO phase provides abundant surface oxygen and hydroxyl groups for reaction initiation. The Ni/NiO interface creates a synergistic effect: electron donation from Ni^0^ to Ni^2+^ shifts the d‐band center and optimizes the adsorption energies of OER intermediates (OH*, O*, OOH*), reducing the energy barrier for key reaction steps.^[^ ^19^ ^]^ This hybrid electronic environment enables intermediate binding strengths that satisfy the Sabatier principle, thereby lowering the overpotential. Additionally, the NiO phase introduces redox flexibility, allowing interfacial Ni atoms to oscillate between Ni^2+^, Ni^3+^ states during the reaction, which supports multielectron transfer and stabilizes high‐valent intermediates essential for O—O bond formation. Metallic Ni functions as an electron donor and provides key adsorption sites for reaction intermediates and NiO serves as a ligand‐like environment that modulates the electronic structure and stabilizes reactive species. The NiO matrix can withdraw electron density from adjacent Ni atoms, promoting the formation of high‐valent Ni species such as Ni^3+^. This charge redistribution tunes the d‐band center of Ni, optimizing the adsorption energies of key intermediates and also facilitates faster charge transfer across the interface. Thus mixed‐phase boundary introduces interfacial strain and defect sites that further enhance activity and stability. Also, the interface influences bubble dynamics and mass transport, contributing to more efficient gas evolution and continuous catalytic performance. Overall, the metal–ligand synergy in NiO/Ni systems represents how an interfacial engineering can expose superior activity through supportive electronic and structural effects. Thus metal–ligand interface acts as a catalytically active area where the metal provides rapid electron transport and the oxide modulates surface reactivity, resulting in improved activity.

## 4 Electrochemical Analysis

### 4.1 Linear sweep voltammetry (LSV)

**Figure 5:**
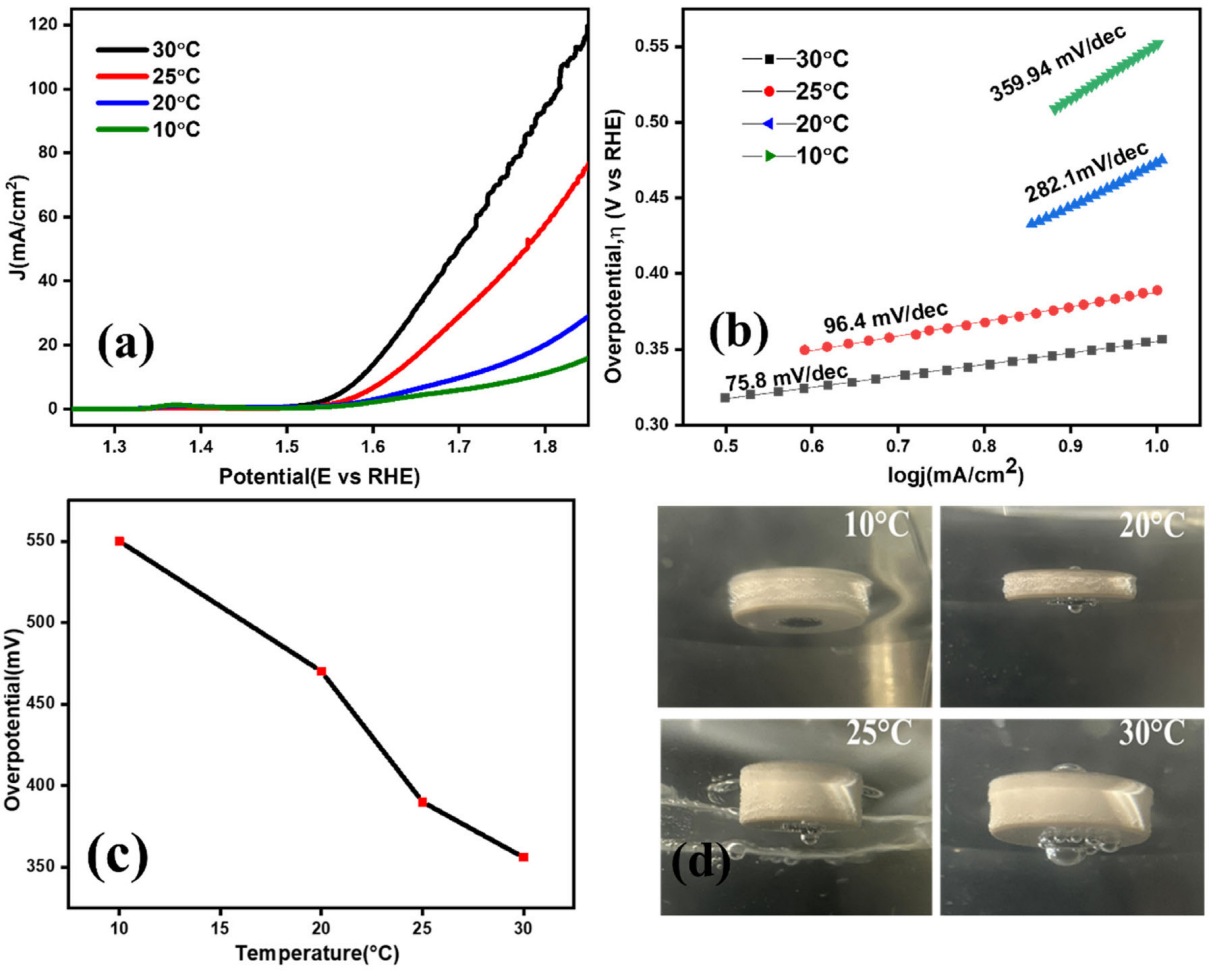
a) LSV curves of NiO/Ni at different electrolyte temperatures, b) Tafel slope corresponding to each temperature, c) plot showing the overpotential‐temperature dependance, and d) oxygen gas formation at different temperatures.

Electrochemical polarization curves with 85% iR (voltage drop) compensation was employed to study the OER activity of the catalyst under different electrolyte temperatures in 1 M KOH, as shown in **Figure** **5**a. LSVs at each temperature were repeated 3 times and approximately the same current density was obtained. Figure 5b shows the Tafel plot corresponding to each temperature. The Tafel slope(b) in Equation (7) calculated provides information about the reaction kinetics and the efficiency of the electron transfer. A lower Tafel slope indicates more efficient catalytic behavior, further supporting the improved performance of the NiO/Ni catalyst at elevated temperatures. The LSV curves demonstrate the effect of temperature on the electrochemical performance of the catalyst, from 10 to 30 °C. As the temperature increases, the current density also rises, indicating improved catalytic activity. This enhancement can be due to the increased thermal energy, which facilitates faster reaction kinetics and better electron transfer at the catalyst's surface. The overpotential required to obtain a current density of 10 mA/cm^2^ were recorded at 550, 470, 390, and 356 mV for 10, 20, 25, and 30 °C, respectively. A lower overpotential at higher temperatures indicates more efficient OER activity. Specifically, at 30 °C, the catalyst exhibited the lowest overpotential of 356 mV, suggesting optimal catalytic performance at this temperature. Figure 5c illustrates decreasing trend in overpotential values with increasing electrolyte temperature, highlighting the enhanced electron transfer capability at elevated temperatures. This trend indicates that elevated temperature promote more efficient catalytic reactions by reducing the energy barrier for the OER process. The shift of the LSV curve toward lower potential as the temperature rises confirms the improved catalytic activity of the NiO/Ni catalyst. The formation of oxygen bubbles at the electrode tip was more at higher temperatures and less at lower temperatures as shown in Figure 5d indicates a better OER activity on lower overpotential. At lower temperatures, the reaction kinetics slow down, leading to higher overpotential. At lower temperatures, the ions in the electrolyte move more slowly, thereby hindering the overall reaction rate. Additionally, the intrinsic reaction rate decreases, making the OER process less efficient. Furthermore, the catalytic activity of NiO may be less effective at low temperatures, requiring higher overpotentials to drive the reaction. The conversion of Ni^2+^ to Ni^3+^ is confirmed by an oxidation peak observed versus RHE potential. Ni^3+^ plays a key role in the OER due to its ability to facilitate proton‐coupled electron transfer. This property enables Ni^3+^ to effectively participate in the transfer of protons and electrons, thereby accelerating the OER process. The enhanced catalytic activity at higher temperatures was attributed to the presence and activity of Ni^3+^. The charge of metals directly influences the electronic structure, binding strength to reactants, and reaction pathway of catalytic activity. Metals capable of exhibiting both stable and variable oxidation states can serve as effective electrocatalysts for OER.^[^ ^66^ ^]^ Water splitting by transition metal‐based catalysts accumulate charged species through changes in their oxidation state.^[^ ^67^ ^,^ ^68^ ^]^ These high‐valent metal species (e.g., Ni^3+^) as electron‐deficient centers facilitate the oxidation of water molecules.^[^ ^69^ ^,^ ^70^ ^]^ This redox cycle enables the catalyst to repeatedly accept and donate electrons, driving the multi‐electron transfer steps required for OER. The ability of the catalyst to stabilize and utilize these high‐oxidation‐state intermediates is critical for lowering the overpotential and enhancing reaction kinetics.^[^ ^71^ ^]^ Transition metals like Nickel undergo oxidation to form high‐valent species like NiOOH, species widely recognized as active sites for OER.^[^ ^72^ ^,^ ^73^ ^]^ Ni active centers along with NiO and special e_g_ orbitals, lowers the activation energy barriers facilitates OER.^[^ ^19^ ^]^ Thus, strategies that promote the formation of high‐oxidation‐state intermediates can lower the onset potential and accelerate reaction kinetics, offering a promising route to improved electrocatalytic performance.^[^ ^74^ ^]^

Tafel slope(b) is a key parameter that quantifies the relationship between the overpotential (*η*) and the logarithm of the current density (log j) in an electrochemical reaction. It is a component of the Tafel equation,

(7) η=a+b.log|j|

that describes the behavior of electrochemical reactions in the Tafel region. Where *η* is the overpotential of the current density, and b denotes the Tafel slope, where b=2.303RT/αF, which represents the kinetic behavior of the OER reaction occurring at the electrode/electrolyte interface. A lower tafel slope value represents an effective charge transfer, which is explained from the inverse relation of slope to charge transfer coefficient *α*. The electrocatalytic performance of the NiO/Ni catalyst was evaluated via Tafel slope analysis at varying temperatures, revealing temperature‐dependent kinetic behavior. From the figure, the tafel slope values decrease with increasing temperature, indicating enhanced reaction kinetics and reduced activation barriers. Theoretical tafel slope values of 120, 30, and 20 mV dec^−1^ at room temperature of 25 °C, respectively corresponds to different RDSs of OER mentioned as in Equation (1)–(4).^[^ ^75^ ^]^

At 30 °C, the Tafel slope decreases to 75.8 mV dec^−1^ compared to other temperatures, indicating enhanced charge transfer. This suggests that, besides electron transfer, a chemical step such as O–H bond cleavage becomes more influential in leading the RDS at elevated temperatures.^[^ ^76^ ^]^ This lower value suggests that the catalyst surface is largely covered by early‐stage intermediates, such as *OH.^[^ ^77^ ^]^ Applied potential largely alters the equilibria of these earlier intermediates rather than directly accelerating the RDS, resulting lowering of slope. This implies that charge transfer is relatively facile at 30 °C, while a successive chemical transformation after electron transfer (O—H bond breaking) or coverage dynamics increasingly limit the overall reaction rate. At 25 °C, a slope of 96.4 mV dec^−1^ is observed, which lies between the theoretical values for OH^−^ adsorption (120 mV dec^−1^) and O—H bond breaking (30 mV dec^−1^), suggesting a mixed RDS involving both processes.^[^ ^78^ ^]^ The intermediate formed immediately before the RDS becomes more significant on the surface, with its coverage fraction rising relative to early‐stage species. As a result, the system gradually shifts toward a situation where the step preceding the RDS controls the kinetics more directly. At 10 and 20 °C, the Tafel slope reaches 359.9 mV dec^−1^ and 282.1 mV dec^−1^, respectively, suggesting a slow kinetics likely dominated with high energy barriers and poor charge transfer. This suppressed kinetics at low temperature leading to a very small exchange current density (*j*_0_), which causes the surface intermediates to respond sluggishly to potential, and the higher values reflecting nonideal kinetics possibly due to surface passivation, bubble accumulation, or limited conductivity. These values also indicate that the reaction is hindered by mass transport or interfacial limitations. These results align with the multistep nature of the OER, where the RDS can shift with temperature. The observed decrease in Tafel slope with increasing temperature reflects a compensation effect, where thermal activation facilitates intermediate formation and charge transfer, effectively lowering the kinetic barrier. The lowest Tafel slope of 75.8 mV dec^−1^ at 30 °C highlights the superior OER activity of the NiO/Ni catalyst under mild thermal conditions. This value approaches the ideal range for mixed‐step kinetics and suggests that the catalyst benefits from synergistic effects between Ni and NiO phases, possibly enhancing OH^−^ adsorption and facilitating O—H bond cleavage. This indicates that temperature variation has a strong impact on how the reaction proceeds, influencing the rate determining step and overall reaction kinetics. High‐performing electrodes (with a lower overpotential) were found to affect the RDSs in this temperature range. The lower Tafel slope of NiO/Ni at 30 °C suggests improved reaction kinetics, indicating that this temperature is more favorable for NiO/Ni's OER performance.

#### 4.1.1 Cyclic voltammetery (CV)

**Figure 6:**
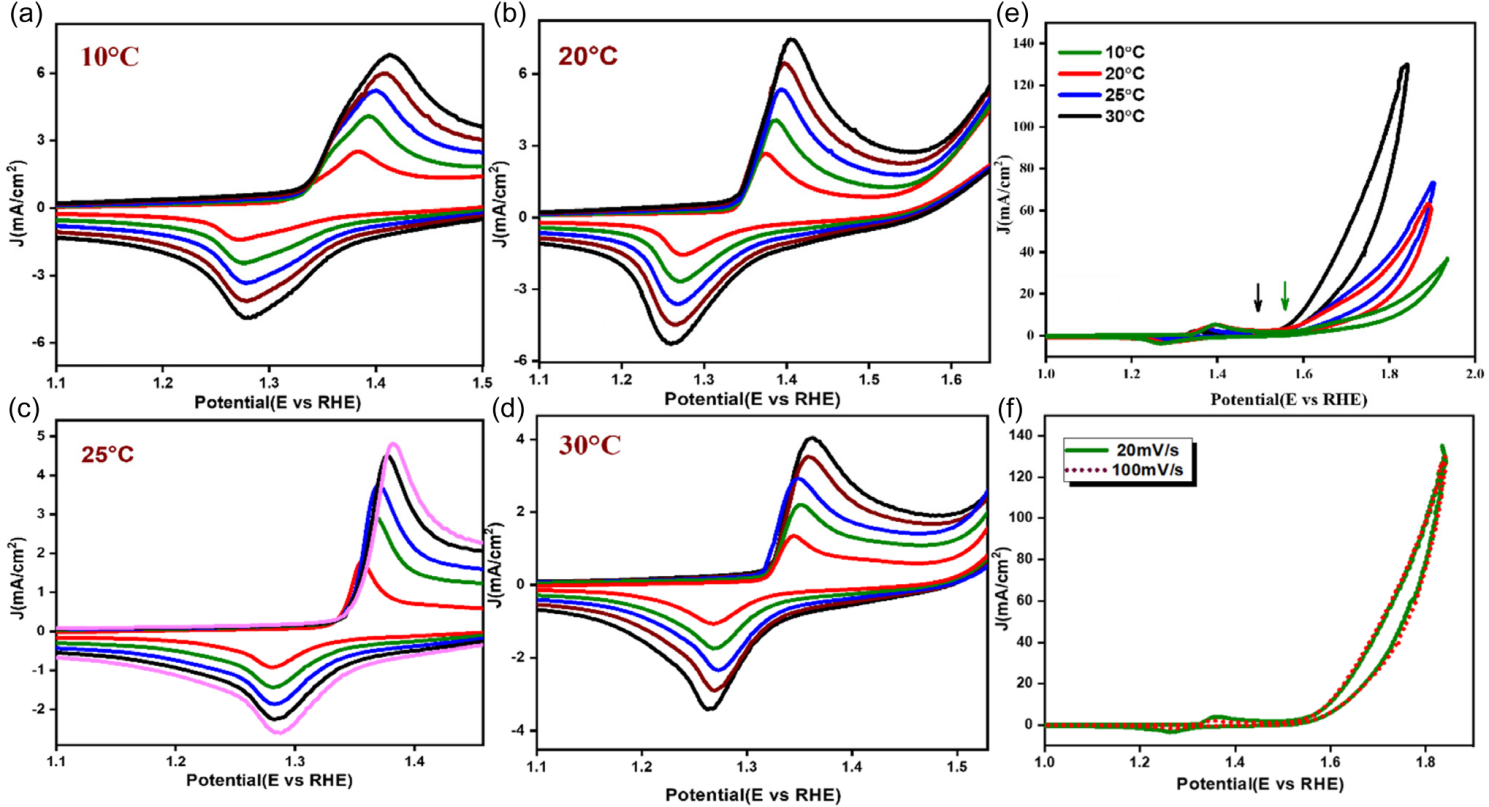
a–d) Enlarged redox portion of the CV curves at different scan rates (20–200 mV s^−1^), e) CV curves at 40 mV s^−1^ under different temperatures showing onset potential shift, and f) CV curves at 30 °C under a low and high scan rate.

To study the redox properties and electrochemical behavior of materials under different electrolyte temperatures, CV was performed on the synthesized catalyst structures with both Ni and NiO phases present, under 20–100 mV/s scan rates and temperatures from 10 °C, 20 °C, 25 °C to 30 °C. The CV curves provide information about the charge transfer kinetics, electrode stability, and overall performance of NiO/Ni in OER. Two defined peaks, anodic and cathodic at the potential values 1.45, and 1.34 V (vs. RHE), respectively observed, indicates the Ni ion's redox transition due to noncapacitive faradaic behavior and explains the redox peaks for Ni(II) to Ni(III) (oxidation of Ni^II^(OH)_2_ to Ni^III^OOH).^[^ ^79^ ^]^ NiOOH species on the surface have been identified as active locations for OER in Ni‐based oxide.^[^ ^80^ ^,^ ^81^ ^]^ A series of enlarged redox portions of the CV curves of the catalyst are plotted under different scan rates from 20, 40, 60, 80 and 100 mV s^−1^ at each temperature as shown in **Figure** **6**a–d. The redox reaction of nickel oxide (NiO) in an alkaline solution proceeds as follows

(8) $$\mathrm{NiO}+{\mathrm{OH}}^{-}\to \mathrm{NiOOH}+e^{-}$$

Subsequently, the nickel oxyhydroxide (NiOOH) formed in the reaction participates in the following reversible redox process

(9) $$\mathrm{NiOOH}+e^{-}↔\mathrm{NiO}+{\mathrm{OH}}^{-}$$

The reactant molecule's kinetic energy increases as the temperature rises. This increased kinetic energy makes it easier for the reactants to overcome the activation energy barrier, making the oxidation reaction (anodic process) more favorable at low potentials. Hence, a negative shift in the anodic peak potential is observed from Figure 6a–d, indicating kinetically favored oxidation of Ni(OH)_2_ and generation of active NiOOH sites at a lower potential.^[^ ^42^ ^]^

As the temperature of the electrolyte increases from 10 to 30 °C, a noticeable shift of potential is observed, which moves to a lower potential than 1.4 V, indicates a reduction in overpotential on higher temperatures. An increase in temperature significantly shifted the onset of the OER toward less positive potential as shown in Figure 6e, at a scan rate of 40 mV/s under the four electrolyte temperatures. The arrow mark in the figure shows the shift of the onset potential from 1.43 (10 °C) to 1.37 V (30 °C), indicates an increased OER at higher temperature. An increase in the current density value is observed at each temperature as the scan rate increases; Figure 6f shows the current density change at 30 °C under a lower and higher scan rate. Higher temperatures enhance the kinetics of electrochemical processes. This means that the rate at which the oxidation process occurs increases with temperature, contributing to the observed shift. At elevated temperatures, the adsorption of reactive species (like OH^−^) on the electrode surface becomes more efficient, further promoting the oxidation process. Thermally induced exposure of active sites is an important phenomenon that can significantly impact the electrochemical performance of materials.^[^ ^82^ ^]^ The increased intensities of the anodic peaks suggest that the number of active centers is increasing with increasing temperature. The initial increase in temperature exposes more active sites and enhances reaction kinetics and anodic peak current, but further temperature increase might lead to a decrease in the effective active sites with an observed shift to lower potential as observed in the redox CV curve portion in Figure 6c,d. This decrease in the anodic current can be due to the covering a portion of active sites by continuous O_2_ bubble formation due to enhanced OER activity at higher temperature, which is shown in the Figure 5d as bubbles and cause the mass transport limitations.^[^ ^83^ ^]^

#### 4.1.2 Electrochemically Active Surface Area (ECSA) and Stability

**Figure 7:**
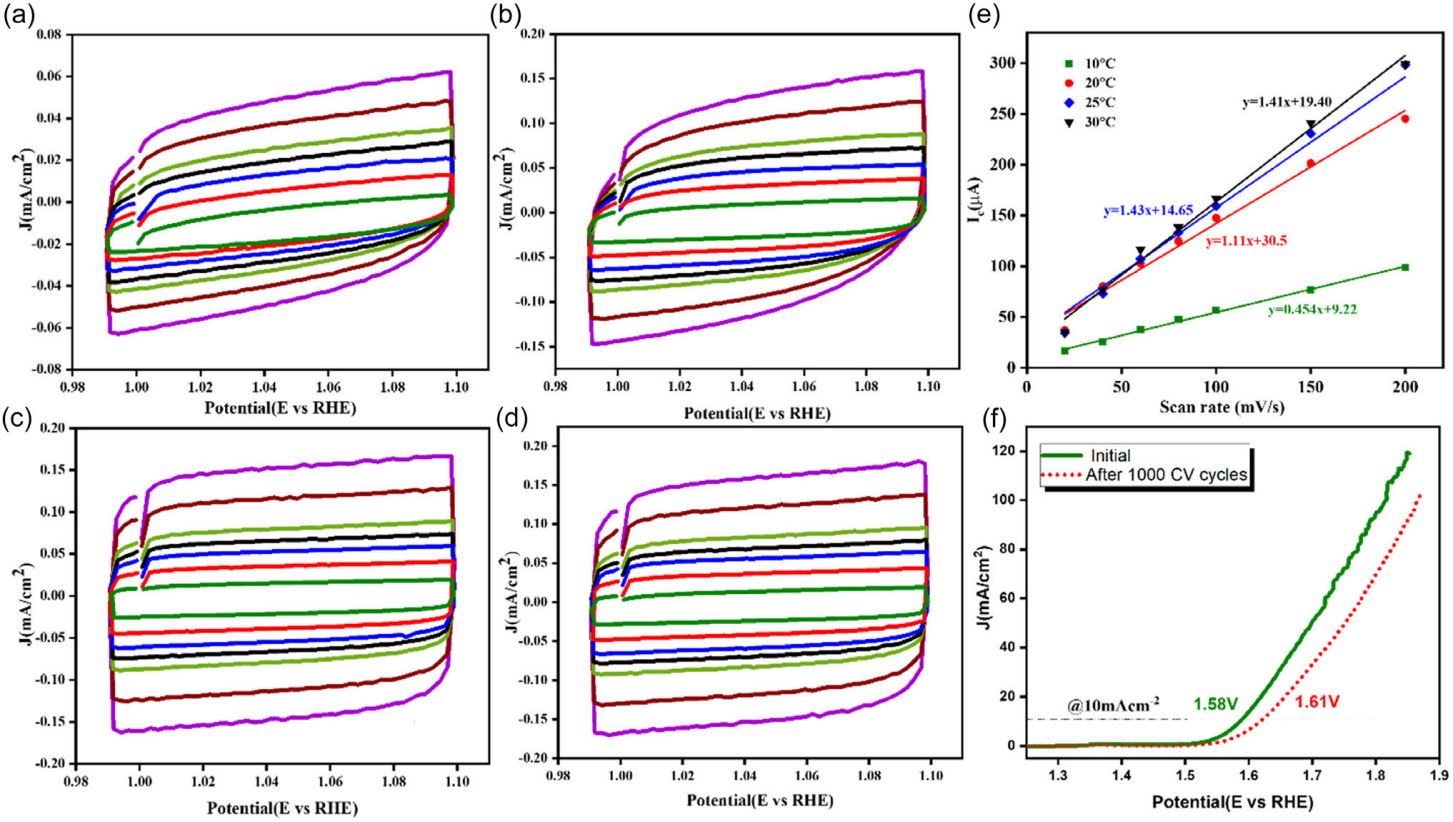
a–d) CV curves of NiO/Ni at different scan rates, e) obtained double‐layer capacitance (*C*_dl_), and f) polarization curve recorded before and after 1000 cycles of CV stability run in 1 M KOH solution.

CV curves of the ECSA measurements under different temperatures at various scan rates (20, 40, 60, 80, 100, 150, and 200 mV/s) are shown in **Figure** **7**a–d and the obtained *C*_dl_ graph in 7e. The determined linear slope values were 0.454, 1.11, 1.43, and 1.44 for 10, 20, 25, and 30 °C, respectively. The significant jump from 10 to 20 °C suggests a major increase in the active surface area or improved wettability of the electrode. Additionally, an increase in *C*_dl_ from 20 to 30 °C is observed, but minimal, indicating a potential saturation effect where further temperature rise does not contribute significantly to increasing ECSA. More active sites are available for electrochemical reactions when the ECSA is large, which boosts catalytic activity^[^ ^84^ ^]^ **.** Up to 20 °C, increased ion mobility, and structural changes in the catalyst exposed more active sites, leading to higher ECSA and enhanced OER activity. However, beyond 25 °C, while more active sites may still be present, the rapid OER kinetics result in increased oxygen bubble formation, which can temporarily block these sites, limiting further improvement in *C*_dl_. Additionally, reduced bubble detachment efficiency restricts ion transport and charge transfer, contributing to the plateau in *C*_dl_. Despite this, the overall reaction kinetics continue to improve at higher temperatures due to lower activation energy and faster electron transfer.

The long‐term electrochemical durability of the catalyst was assessed by comparing the linear sweep voltammetry (LSV) curves recorded before and after 1000 continuous CV cycles in 1.0 M KOH at 30 °C. As shown in Figure 7f, the initial polarization curve (green solid line) exhibits superior catalytic performance with a lower overpotential at the benchmark current density of 10 mA/cm^2^, achieved at ≈1.58 V vs. RHE. After 1000 CV cycles (red dashed line), the polarization curve exhibits a positive shift in potential, requiring ≈1.61 V vs. RHE to reach 10 mA/cm^2^. This shift of about 30 mV indicates a slight decrease in catalytic activity, indicating good electrochemical resilience with only a minor performance loss, can be due to surface reconstruction, active site leaching, or partial oxidation of active species under prolonged electrochemical stress. The minimal increase in overpotential and preserved shape of the LSV curve suggests that the catalyst structure remains largely intact and capable of sustaining water oxidation reactions over extended periods.

**Figure 8:**
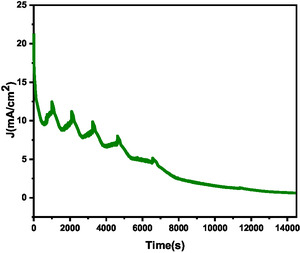
Chronoamperometry (CA) test performed at 30 °C at a potential of 1.6 V (RHE).

CA at 30 °C (**Figure** **8**) under potential 1.6 V exhibited an initially high current density, confirming its favorable oxygen evolution kinetics at elevated temperature, which enhances ionic conductivity and promotes efficient charge transfer. During prolonged operation, periodic current fluctuations were observed, which correspond to bubble nucleation and detachment at the catalyst surface. When bubbles form and detach from the electrode surface, they lower the overpotential. This shows they play a role by temporarily storing oxygen and reducing its buildup near the electrode.^[^ ^85^ ^]^ As a result, the bubbles help keep the reaction area clear and improve the movement of reactants. Elevated temperatures enhance reaction kinetics, resulting in higher gas generation rates. Bubbles form more rapidly and can reach detachment size sooner, increasing the frequency of detachment events. This dynamic interaction modulates the electrochemical interface, temporarily altering the effective active area and mass transport conditions. Also, minor physical movement of the catalyst layer due to the mechanical force of gas release caused by continuous detachment and reattachment, further contributing to the observed current behavior. Following this, the system transitions to a more stable region, where current density gradually declines and plateaus, likely due to surface restructuring, reactant depletion. In literature, it explains the challenges of Ni‐based nanomaterials in achieving optimal activity and long‐term stability. This limitation arises from insufficient exposure of active sites and restricted interaction with electrolyte, often caused by severe particle aggregation or structural degradation. These issues are particularly pronounced in ultra‐small nanoclusters, where high surface reactivity accelerates oxygen gas evolution, leading to mechanical instability and compromised performance.^[^ ^86^ ^]^ These initial results highlight the temperature‐dependent modulation of catalytic performance. Detailed studies addressing thermal stability and long‐term deactivation behavior are currently in progress.

## 5 Conclusion

This work studies the impact of electrolyte temperature on the OER activity of NiO/Ni catalyst and proves that even slight temperature variations can significantly impact catalytic activity. Elevated temperatures enhance performance by reducing overpotential (from 550  to 356 mV) and Tafel slope reduced to 75.8 mV dec^−1^, while increasing current density, making the catalyst more effective for practical applications. The increase in double‐layer capacitance (*C*_dl_) with temperature indicates a larger ECSA, which in turn increases the availability of active sites for the reaction and contributes to the overall improvement in catalytic activity. The morphology and synthesis method play crucial roles in facilitating smooth electron transfer, further enhancing performance. At higher temperatures, the reaction rate improves as more energy is available to overcome activation barriers, leading to improved catalytic efficiency. This is reflected in the decrease in overpotential, which indicates a lower energy requirement to drive the OER at elevated temperatures. However, oxygen bubble formation remains a key challenge, particularly at 25 and 30 °C, because it can block active sites and hinder mass transport, limiting further improvement in performance despite the increased ECSA. This highlights the importance of addressing bubble‐related restrictions for future catalyst design and optimization. Despite these challenges, the results confirm that temperature‐dependent analysis is essential for fine‐tuning OER catalysts. This work underscores the potential of nickel‐based catalysts as active, cost‐effective alternatives for sustainable energy applications, highlighting the need to focus on future studies on mass transport limitations for optimal electrochemical activity.

## Conflict of Interest

The authors declare no conflict of interest.

## Author Contributions

**Easwari Padma Kumari**: data curation (lead); formal analysis (lead); investigation (lead); writing—original draft (lead). **Anand Kumar**: funding acquisition (equal); supervision (equal); writing—review & editing (supporting). **Faris Tarlochan**: supervision (supporting); writing—review & editing (supporting). **Mohammed J. Al‐Marri**: funding acquisition (equal); supervision (supporting); writing—review & editing (supporting).

## Data Availability

The data that support the findings of this study are available from the corresponding author upon reasonable request.
